# Change in the Pathologic Supraspinatus: A Three-Dimensional Model of Fiber Bundle Architecture within Anterior and Posterior Regions

**DOI:** 10.1155/2015/564825

**Published:** 2015-08-27

**Authors:** Soo Y. Kim, Rohit Sachdeva, Zi Li, Dongwoon Lee, Benjamin W. C. Rosser

**Affiliations:** ^1^School of Physical Therapy, College of Medicine, University of Saskatchewan, Saskatoon, SK, Canada S7N 0W3; ^2^Department of Surgery, University of Toronto, Toronto, ON, Canada M5T 1P5; ^3^Department of Computer Science, University of Toronto, Toronto, ON, Canada M5S 3G4; ^4^Department of Anatomy and Cell Biology, College of Medicine, University of Saskatchewan, Saskatoon, SK, Canada S7N 5E5

## Abstract

Supraspinatus tendon tears are common and lead to changes in the muscle architecture. To date, these changes have not been investigated for the distinct regions and parts of the pathologic supraspinatus. The purpose of this study was to create a novel three-dimensional (3D) model of the muscle architecture throughout the supraspinatus and to compare the architecture between muscle regions and parts in relation to tear severity. Twelve cadaveric specimens with varying degrees of tendon tears were used. Three-dimensional coordinates of fiber bundles were collected *in situ* using serial dissection and digitization. Data were reconstructed and modeled in 3D using Maya. Fiber bundle length (FBL) and pennation angle (PA) were computed and analyzed. FBL was significantly shorter in specimens with large retracted tears compared to smaller tears, with the deeper fibers being significantly shorter than other parts in the anterior region. PA was significantly greater in specimens with large retracted tears, with the superficial fibers often demonstrating the largest PA. The posterior region was absent in two specimens with extensive tears. Architectural changes associated with tendon tears affect the regions and varying depths of supraspinatus differently. The results provide important insights on residual function of the pathologic muscle, and the 3D model includes detailed data that can be used in future modeling studies.

## 1. Introduction

Supraspinatus tendon tears of the rotator cuff are associated with changes in both the tendon and muscle. Fraying and thinning of the lateral aspect of the tendon occur with full-thickness tears [1, 2]. Muscular inhibition and disuse due to pain can lead to changes in the muscle. A decrease in muscle volume and fat infiltration can occur with large tendon tears [3–5]. The musculotendinous unit of the supraspinatus can also retract medially altering the length of fiber bundles [6, 7]. The alignment of fiber bundles relative to the axis of pull or line of force which is known as the pennation angle (PA) can also change as a result of retraction [8].

The function of a muscle is directly correlated with its architecture. Among the architectural parameters of skeletal muscles, fiber bundle length (FBL) is known to be the most important as it is proportional to muscle excursion and the velocity of contraction [9, 10]. A direct linear relationship has been found between muscle length and force of isometric contraction [11]. Thus, a change in FBL can affect the optimal range and speed at which a muscle contracts [12]. In pennated muscles, only a component of the muscle fibers' force is projected onto the line of force; thus a change in PA will also impact the force-producing capabilities [13].

The muscle and tendon architecture of the supraspinatus is complex. The normal muscle has two main regions, anterior and posterior [14–18], which have been found to be functionally distinct [19–21]. The anterior region accounts for 75–86% of the muscle volume and its pennated fiber bundles attach laterally to the anterior tendon [14, 16]. It produces the majority of force for the muscle [14, 16]. The posterior region is substantially smaller in volume and partially lies deep to the anterior. The parallel fiber bundles attach laterally to the posterior tendon [14, 16]. Based on its architecture, dynamic FBL changes with shoulder movements [19], fiber type composition [20], and innervation pattern [21], the posterior region is thought to play an important role in adjusting tension on the rotator cuff. Within each region there are three distinct parts, superficial, middle, and deep, based on the lateral attachment sites onto the tendon and fiber bundle orientation [16].

To date, the fiber bundle architecture of the pathologic supraspinatus has not been investigated throughout the muscle volume. In previous investigations, fiber bundle measurements were taken from the superficial surface of the muscle, not accounting for the different regions and parts of the muscle [6, 7]. Since the length of fiber bundles and PA are directly related to skeletal muscle function [22], it is important to quantify these parameters for both the anterior and posterior regions of the pathologic supraspinatus. In addition, given that architectural changes are considered to be the most important pathophysiological consequence of tendon tears and a critical factor in the success of tendon repair surgery, a thorough understanding of these parameters is needed [23].

A robust fiber bundle architecture database of the pathologic supraspinatus can be used to advance three-dimensional (3D) musculoskeletal computer models of the shoulder. 3D modeling is a powerful tool for analyzing the biomechanics underlying normal and pathological movements, particularly in complex systems such as the shoulder [24, 25]. Finite-element muscle models can provide detailed information about the distribution of strain within a muscle and the transmission of force [24]. These models, which are dependent on accurate fiber bundle data, can be used to make clinically relevant predictions about the functional deficits caused by rotator cuff tendon tears and the functional outcomes following surgery and rehabilitation [26, 27].

The purpose of this study was to investigate and model the muscle architecture throughout the volume of the supraspinatus using cadaveric specimens with varying degrees of tendon tears. It was hypothesized that fiber bundle architecture would differ between the anterior and posterior regions and their respective parts, superficial, middle, and deep, and that the architectural changes would be associated with the degree of tendon pathology.

## 2. Materials and Methods

### 2.1. Specimens

Twelve formalin embalmed cadaveric shoulder specimens (3 males, 9 females) with evidence of supraspinatus tendon pathology, that is, partial thickness or full-thickness tears, were used. Mean age was 82.1 ± 10.8 years with a range of 64–95. Ethics approval was obtained from the Biomedical Research Ethics Board, University of Saskatchewan (Bio#11-77).

To expose the muscle and determine the presence of supraspinatus tendon pathology all overlying soft tissues (skin, fascia, trapezius, and deltoid) were removed. The clavicle and lateral aspect of the acromion were also removed to allow full visualization of the rotator cuff tendons. Specimens with evidence of shoulder surgery or gross bony deformities were not used.

Specimens were placed into one of three categories based on the degree of tendon tear of the supraspinatus: (A) partial thickness tear; (B) full-thickness tear with no tendon retraction; (C) full-thickness tear with tendon retraction. A tear was deemed as having tendon retraction when the tear involved the entire extent (width) of the supraspinatus tendon in the sagittal plane. In specimens with full-thickness tendon tears (“B” and “C”), coronal and sagittal dimensions of the tear were measured using a digital caliper (Traceable ISO 17025 Calibrated, Fisher Scientific, Nepean, ON, Canada) and recorded.

### 2.2. Dissection and Digitization

**Figure 1:**
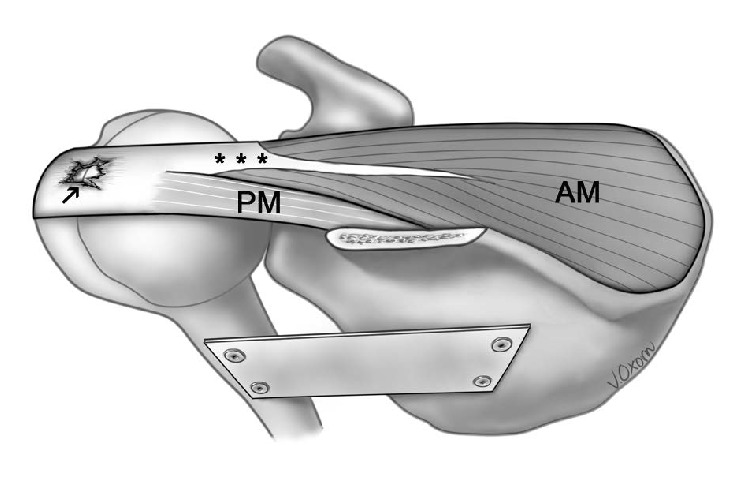
Supraspinatus with full-thickness tendon tear with no retraction (specimen representative of category B). Superior view of the middle part of the muscle belly with acromion removed. Fiber bundles of the superficial parts of anterior and posterior regions have been removed. Specimen stabilized with metal plate. Anterior part of supraspinatus tendon represented by ∗∗∗; middle part of anterior region (AM); middle part of posterior region (PM). Fiber bundles of deep parts lie deep to the AM and PM. Arrow (↑) points to a full-thickness tear illustrated on the supraspinatus tendon.

Each of the 12 specimens was digitized. Previously developed digitization protocols for human skeletal muscles were adapted for this study [16, 28]. The glenohumeral joint was stabilized in 0° of abduction, flexion, and lateral rotation with a metal plate screwed to the humerus and scapula (Figure 1). The lateral aspect of the scapular spine, coracoid process, and greater tubercle were selected as reference points and demarcated with screws. These reference points were used in the modeling process and assisted in reconstructing the specimen in 3D. Specimens were then clamped into a securely mounted vice.

The periphery of the supraspinatus tendon was outlined with small dots 2 mm apart using a paint pen. Next, each point was digitized using a Microscribe G2X Digitizer (Immersion Corporation, San Jose, CA, USA). Following this, the division between the anterior and posterior regions on the superficial surface of the muscle as defined by Kim et al. [16] was identified and marked with small pins (3 mm in length). The anterior region of the supraspinatus muscle belly was serially dissected and digitized* in situ* first. Starting with the most superficial layer, 10–60 fiber bundles were identified. Each fiber bundle was then digitized using 10–20 sequential sites, beginning at the medial attachment site and ending at the lateral. Once the entire layer had been digitized, fiber bundles were carefully removed to expose the underlying fascicles about 1-2 mm deeper. The periphery of the tendon was digitized whenever the tendon shape was found to change which was approximately at every 3–5 mm of the muscle's depth. Once the entire anterior region had been digitized, the posterior region was serially dissected and digitized as outlined above.

### 2.3. Modeling

**Figure 2:**
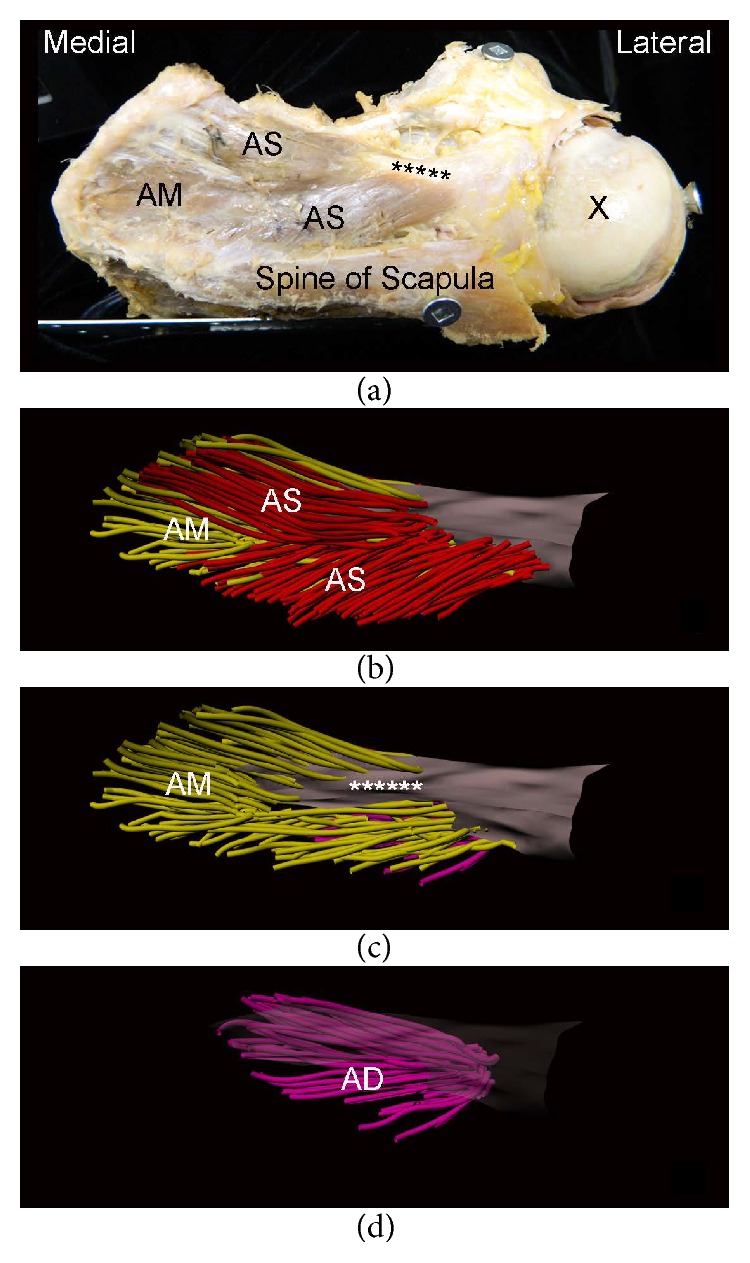
Retracted full-thickness tendon tear of supraspinatus without a distinguishable posterior region modelled throughout its volume in 3D (specimen from category C). (a) Superior view of specimen with lateral aspect of spine of scapula removed; superficial part of anterior region (AS); middle part of anterior region (AM); ∗∗∗ intramuscular portion of anterior supraspinatus tendon. Metal plate (bottom left) was used to stabilize the specimen and screws (scapular spine, coracoid process, and greater tubercle) were used in modeling process. (b) Computer model of anterior region including AS (red fibers) and AM (yellow fibers). (c) AM; AS not shown; (d) deep part of anterior region (AD) (pink fibers); AS, AM, and tendon are not shown.

Digitized data were exported to Autodesk Maya 2009 (Autodesk, San Jose, CA, USA) and reconstructed in 3D using plug-ins developed in the laboratory. Fiber bundles and their attachment onto the tendon could be clearly visualized volumetrically using the model. Architecturally distinct regions and parts as defined by Kim et al. [16] were identified and color coded (Figure 2).

### 2.4. Data Analysis

Fiber bundle lengths and PA were computed with algorithms used in previously published work [16, 29, 30]. For a detailed description of computational methods the reader is referred to Lee et al. [29, 30]. Digitized fiber bundles were first reconstructed into an interpolating cubic Catmull-Rom spline. Using arc-length parameterisation, digitized points were then resampled to make the curve representation uniform. FBL was approximated as an entire arc-length of the curve [29]. In the present study, PA is defined as an angle between the fiber bundle orientation and the line of force. The fiber bundle orientation was estimated by a tangent vector along the curve. The tangent vectors at the lateral and medial attachment sites were calculated as average derivatives of the curve over the lateral and medial regions, respectively [29, 30]. Hence, two angles were computed, lateral PA and medial PA. The line of force was determined as a vector best approximating the axis of the intramuscular tendon. 

Statistical analysis was carried out using SPSS (version 18.0, Chicago, IL, USA). All architectural parameters for the anterior and posterior regions and their distinct parts were characterized with descriptive statistics (median and minimum-maximum values). Mann-Whitney* U* tests were used to compare median tear dimensions between specimen categories B and C and architectural parameters between categories of A and B of the posterior region. The Kruskal-Wallis test followed by pairwise comparisons (Mann-Whitney* U* tests) was used to compare median age of specimens and architectural parameters between the three tear categories and between superficial, middle, and deep parts. Significance was accepted at *P* < 0.05 with Bonferroni adjustments made where appropriate (0.05/3 = 0.0167).

## 3. Results

### 3.1. Tendon Morphology

Within each of the three tendon tear categories, there were four specimens. The largest diameters of the tear in the coronal and sagittal planes, measured in specimens of categories B and C, are presented in Table 1. The median tear dimensions in category C (2.96 cm for coronal plane; 3.72 cm for sagittal plane) were significantly larger than those in category B (1.58 cm for coronal plane; 1.44 cm for sagittal plane) for both planes (*P* = 0.020 for coronal and *P* = 0.021 for sagittal). All specimens in category C also had a tear of the infraspinatus and subscapularis tendons. There was no difference in the median age of specimens between categories.

### 3.2. Muscle Morphology

#### 3.2.1. Anterior Region

Table 2 provides a summary of the architectural parameters of the anterior region as a whole. In all specimens an anterior region was present (Figure 1). Median FBL significantly differed between the three tear categories (*P* < 0.001). Specimens of category C had the shortest fibers. Median lateral PA in category C was significantly larger than categories A and B (*P* < 0.001). Median medial PA significantly differed between all tear categories (*P* < 0.001), with the largest PA found in category C.

Table 3 provides a summary of median FBL values of the superficial, middle, and deep parts within the anterior region. Median FBL was significantly different between all the parts in each tear category. The significance level was *P* < 0.001 except between the superficial and deep in category B and the middle and deep in category C, which was *P* = 0.001. The middle and deep parts were shorter than the superficial in all categories.

Tables 4 and 5 provide a summary of median lateral and medial PA values of the superficial, middle, and deep parts within the anterior region. Median lateral PA of the superficial part was significantly larger than the middle (*P* < 0.001) in category B. No statistical difference was found between the superficial and deep (*P* = 0.022) and middle and deep (*P* = 0.071). No statistical difference was found between parts in category A (*P* = 0.167) or category C (*P* = 0.274). Median medial PA of the superficial part was significantly larger than the middle and deep (*P* < 0.001) in all tear categories. No statistical difference was found between the middle and deep (*P* = 0.163) in category A. In categories B and C, median medial PA was significantly different between all parts (*P* < 0.001).

#### 3.2.2. Posterior Region

Architectural parameters for the posterior region as a whole are summarized in Table 2. In two specimens of category C a distinct posterior region was absent (Figure 2). Due to the reduced sample size in category C, statistical analysis of these specimens was not carried out. Median FBL significantly differed between categories A and B (*P* < 0.001). Median lateral PA significantly differed between categories A and B (*P* = 0.013). Median lateral PA in category A was larger than category B (*P* = 0.013). Median medial PA in category A was significantly larger than that of category B (*P* < 0.001).

Median FBL values for the superficial, middle, and deep parts within the posterior region are summarized in Table 3. In category A, median FBL of the middle part was significantly longer than that of the superficial and deep (*P* < 0.001). No statistical difference was found between the superficial and deep (*P* = 0.699). In category B, no difference was found between parts (*P* = 0.114).

A summary of median lateral and medial PAs of the superficial, middle, and deep parts within the posterior region can be found in Tables 4 and 5. Median lateral PA of the superficial and middle parts was significantly larger than the deep (*P* < 0.001) in category A. No statistical difference was found between the superficial and middle (*P* = 0.238). In category B, median lateral PA significantly differed between all parts (*P* < 0.001) with the largest being in the superficial part. Median medial PA of the superficial part was significantly larger than the middle (*P* < 0.001) in category A. No statistical difference was found between the deep and middle (*P* = 0.021) or superficial (*P* = 0.026). In category B, median medial PA of the superficial part was significantly larger than that of the deep (*P* = 0.002). No statistical difference was found between the middle and deep (*P* = 0.419) or superficial (*P* = 0.047).

## 4. Discussion

This is the first study to investigate and model the fiber bundle architecture of the pathologic supraspinatus throughout the muscle volume including the anterior and posterior regions and their respective parts. We demonstrate that significant changes in architecture occur with rotator cuff tendon pathology and these changes are not uniform for the anterior and posterior regions of supraspinatus.

Median FBL of the anterior and posterior regions significantly differed between the tear categories, with a gradual decrease in FBL occurring as the size of the tear increased. Significant shortening of FBL with tendon tears has been reported in two previous studies [6, 7]. In both studies, however, only the anterior region was investigated as per reported lateral attachment of fiber bundles onto the intramuscular tendon. In addition, length measurements were taken from just two to three fibers from superficial surface of the anterior region of each specimen. Based on our model, we know that both anterior and posterior regions undergo significant shortening with tendon tears. Shortening of muscle fibers and tendon retraction are barriers for structural healing following open and arthroscopic tendon repair [31]. When structural healing is not achieved, recovery of strength has been poorer and the glenohumeral joint may be more prone to degenerative changes [31, 32].

In the normal supraspinatus, FBLs within the superficial, middle, and deep parts of the anterior region were found to be uniform [16]. In the present study of the pathologic muscle, however, in general as the severity of the tear increased a progressive shortening of FBLs from the superficial to deep parts was observed. This pattern of FBL change within the volume of the anterior region could be of clinical importance. First, tears on the articular surface of the supraspinatus tendon are two to three times more frequent than bursal-sided tears [33, 34]. Our findings further support this prevalence and suggest tears involving the anterior portion of the supraspinatus tendon propagate from the articular surface toward the bursal surface. Secondly, as a consequence of this progressive shortening starting in the deep part of the muscle, the deeper fibers may undergo greater stretching during tendon repair. To achieve tendon to bone repair, the torn and often retracted musculotendinous unit is mobilized laterally. Overstretching, particularly of the shortened deep fibers, can cause damage and lead to proliferation of noncontractile tissue [33]. Furthermore, the articular side of the supraspinatus tendon has been found to experience more strain compared to the bursal surface under uniaxial loading [35]. These tendon strain patterns may be correlated with the pattern of FBL changes observed in this study.

In the posterior region, the pattern of progressive shortening of FBLs from the superficial to deep parts was not observed. The differences found between the anterior and posterior regions may be related in part to the differences in muscle architecture. For example, the fibers of the anterior region are in a penniform configuration, while in the posterior region they are fusiform.

A decrease in FBL will decrease the absolute active muscle range and maximum contraction velocity [36]. In the torn supraspinatus, sarcomeres have been found to maintain their optimal operating length [7]. Tomioka et al. [7] found no significant difference in sarcomere length of the supraspinatus between the intact and torn tendon specimens examined. Despite this maintenance, a recent study investigating the contractility of muscle fibers sampled from patients with chronic full-thickness tears found a 30% reduction in the maximum isometric force production [37]. The normalized force production was found to be negatively correlated with tear size [37]. The architectural changes documented in our study will compound these force production deficits reported by Mendias et al. [37]. Our data on FBL along with insights of sarcomere lengths changes [7] and muscle fiber contractility [37] can be used in future computer modeling studies to predict the changes in active range and contraction velocity of the pathologic muscle and to simulate the biomechanical effects on the shoulder complex.

An increase in PA with rotator cuff tendon tears has been previously reported with larger and retracted tears being correlated with larger angles [8, 17]. Although direct comparison of our PA values with those from other cadaveric and imaging studies is difficult due to differences in measurement methods, our findings support the general trends reported in these previous studies. In the present study, median lateral and medial PAs of the anterior region were significantly larger in specimens with a retracted tendon. In contrast, the median lateral and medial PAs of the posterior region were significantly larger in specimens with partial tears compared to full-thickness tears. Again, these regional differences may be attributed to differences in muscle architecture and possibly the location of the tendon pathology, that is, articular versus bursal sided and/or anterior versus posterior.

The posterior region of the supraspinatus was present in all normal specimens examined by Kim et al. [16] and Roh et al. [14]. The absence of a distinguishable posterior region in half of the specimens with large retracted tendon tears in the present study raises important clinical questions given the broad lateral attachment of fibers onto the supraspinatus tendon and its distinct function [18–21]. First, what is the functional impact of not having a posterior region in both the unrepaired and repaired tendon states? Since the posterior region is thought to quickly adjust tension on the rotator cuff, preventing buckling of the tendon with dynamic movement [20, 21], the loss of this region can have a significant functional impact. The residual function of the pathological muscle thus needs to be better understood. Expectations and approaches for rehabilitation and surgical repair may also need to be altered when there is loss of the posterior region. Secondly, is loss of the posterior region an eventual change that occurs with chronic tendon tears? It is known that chronicity of the tendon tear is positively related to rotator cuff muscle atrophy [38, 39]. As the volume of the posterior region is significantly smaller than that of the anterior region in the normal muscle [14, 16] even a small amount of atrophy could considerably impact the posterior region. If atrophy or complete loss is indeed found to be an eventual consequence of chronic tears, it would further underscore the importance of early detection of the tear and repair of the tendon. Delayed detection of tears is associated with surgical complications and inferior outcomes [40] and extensive changes to the posterior region may play a role in these problems.

## 5. Conclusions

The fiber bundle architecture of both anterior and posterior regions was investigated and distinct patterns of change were found. Fiber bundle length shortening is associated with the degree of tendon tear with the fibers of the deep part showing the greatest degree of shortening. Pennation angle changes are also related to the degree of tendon tear, with the superficial fibers possibly undergoing greater changes than other parts of the muscle. The posterior region was completely absent in specimens with extensive tendon tears raising several clinically relevant questions that need to be further explored. Since the supraspinatus muscle is an important dynamic stabilizer of the glenohumeral joint and most commonly involved with rotator cuff pathology, a thorough understanding of the muscle changes is essential. It is expected that the model created in this study will be used to advance computer models that can simulate different surgical techniques and rehabilitation protocols. Furthermore, the model can be incorporated with existing shoulder models to be used for biomechanical analysis in different patient populations with supraspinatus tendon pathologies.

## Figures and Tables

**Table 1:** Summary of specimens.

Specimen #	Side	Sex	Age	Tear category	RCT location	Coronal plane (cm)	Sagittal plane (cm)
1460	R	F	67	A	SP	—	—
1463	R	F	95	A	SP	—	—
1429	R	F	64	A	SP	—	—
1425	R	M	89	A	SP	—	—
1458	R	F	73	B	SP	1.88	1.63
1450	R	F	77	B	SP	1.04	2.08
1447	R	F	94	B	SP	1.47	1.52
1447	L	F	94	B	SP	1.40	1.17
1455^*^	R	F	82	C	SP, SSC, ISP	3.33	3.84
1427^*^	R	M	76	C	SP, SSC, ISP	5.0	3.00
1444	R	M	83	C	SP, SSC, ISP	2.59	3.76
1445	L	F	91	C	SP, SSC, ISP	2.59	3.67

^*^Specimens with no posterior region. R: right; L: left; F: female; M: male; RCT: rotator cuff tear; A: partial thickness tear; B: full-thickness tear with no retraction; C: full-thickness tear with retraction of the tendon; SP: supraspinatus; SCC: subscapularis; ISP: infraspinatus; —: not measured.

**Table 2:** Median values of architectural parameters for anterior and posterior regions.

Region of muscle and tear category	*n*	FBL (cm)	PA lat. (degree)	PA med. (degree)
Anterior				
A	4	6.76^a^ (3.21–10.26)	14.95^a∗^ (2.04–45.24)	13.81^a^ (2.17–45.11)
B	4	4.97^b^ (2.59–9.86)	13.91^a∗^ (2.01–46.37)	14.77^b^ (2.02–46.63)
C	4	2.65^c^ (0.54–8.98)	23.02^b^ (2.06–80.38)	24.34^c^ (2.01–89.20)
Posterior				
A	4	5.89^a^ (2.12–8.52)	24.14^a∗∗^ (2.04–38.81)	15.94^a^ (3.78–34.52)
B	4	4.81^b^ (1.48–8.92)	20.61^b∗∗^ (4.42–47.55)	11.98^b^ (2.10–34.68)
C	2	3.02^†^ (1.59–4.76)	31.95^†^ (2.42–55.23)	14.29^†^ (4.83–33.15)

A: partial thickness tendon tear; B: full-thickness tear with no retraction of tendon; C: full-thickness tendon tear with retraction; FBL: fiber bundle length; PA lat.: lateral pennation angle; PA med.: medial pennation angle; ( ) minimum and maximum values. If superscript letters are different, it indicates statistically significant difference of *P* < 0.001 of parameter between tear categories within the same region. ^*^ *P* = 0.100; ^**^ *P* = 0.013; ^†^statistical analysis was not conducted due to sample size.

**Table 3:** Median FBL values for the superficial, middle, and deep parts of the anterior and posterior regions.

Region of muscle and tear category	*n*	FBL (cm)		
		Superficial	Middle	Deep
Anterior				
A	4	7.56^a^ (5.36–10.26)	6.71^b^ (3.21–10.01)	6.24^c^ (3.38–8.68)
B	4	5.58^a∗^ (2.97–9.68)	4.71^b^ (2.59–9.86)	5.16^c∗^ (2.70–9.05)
C	4	2.95^a^ (1.45–8.98)	2.57^b∗^ (1.04–7.19)	2.50^c∗^ (0.54–5.41)
Posterior				
A	4	5.89^a∗∗^ (2.12–8.47)	6.42^b^ (2.79–7.91)	5.85^a∗∗^ (3.19–8.52)
B	4	4.88^a∗∗∗^ (2.13–7.82)	4.98^a∗∗∗^ (1.50–8.92)	4.65^a∗∗∗^ (1.48–8.54)
C	2	2.96^†^ (1.90–4.47)	3.48^†^ (1.93–4.76)	2.80^†^ (1.59–4.59)

A: partial thickness tendon tear; B: full-thickness tear with no retraction of tendon; C: full-thickness tendon tear with retraction; FBL: fiber bundle length; median with ( ) minimum and maximum values. If superscript letters are different, it indicates statistically significant (*P* < 0.001) difference between the superficial, middle, and deep parts of specimens within the same tear category. ^*^ *P* = 0.001; ^**^ *P* = 0.699; ^***^ *P* = 0.114; ^†^statistical analysis was not conducted due to sample size.

**Table 4:** Median lateral PA values for the superficial, middle, and deep parts of the anterior and posterior regions.

Region of muscle and tear category	*n*	PA lat. (degree)		
		Superficial	Middle	Deep
Anterior				
A	4	14.26^a∗^ (2.62–45.24)	15.71^a∗^ (2.04–40.86)	14.15^a∗^ (2.37–35.39)
B	4	15.59^a∗∗^ (2.27–46.37)	12.73^b∗∗^ (2.01–42.70)	14.15^ab∗∗^ (2.89–40.38)
C	4	25.16^a∗∗∗^ (2.06–64.58)	22.92^a∗∗∗^ (2.18–80.38)	21.80^a∗∗∗^ (3.15–74.67)
Posterior				
A	4	26.39^a∗∗∗∗^ (2.04–38.81)	25.60^a∗∗∗∗^ (2.19–36.34)	18.79^b^ (3.92–36.57)
B	4	27.17^a^ (9.86–41.02)	23.43^b^ (4.42–47.55)	17.71^c^ (6.32–32.00)
C	2	38.46^†^ (7.82–55.23)	35.25^†^ (2.42–51.13)	25.17^†^ (2.55–46.18)

A: partial thickness tendon tear; B: full-thickness tear with no retraction of tendon; C: full-thickness tendon tear with retraction; PA lat.: lateral pennation angle; median with ( ) minimum and maximum values. If superscript letters are different, it indicates statistically significant (*P* < 0.001) difference between the superficial, middle, and deep parts of specimens within the same tear category. ^*^ *P* = 0.167; ^**^ *P* = 0.022 superficial and deep, *P* = 0.071 middle and deep; ^***^ *P* = 0.274; ^****^ *P* = 0.238; ^†^statistical analysis was not conducted due to sample size.

**Table 5:** Median medial PA values for the superficial, middle, and deep parts of the anterior and posterior regions.

Region of muscle and tear category	*n*	PA med. (degree)		
		Superficial	Middle	Deep
Anterior				
A	4	15.58^a^ (2.19–29.78)	12.97^b^ (2.20–45.11)	13.87^b∗^ (2.17–30.51)
B	4	19.22^a^ (3.88–44.64)	15.95^b^ (2.02–46.63)	11.52^c^ (2.34–27.95)
C	4	28.40^a^ (2.37–89.20)	22.96^b^ (2.01–85.95)	19.99^c^ (2.17–89.11)
Posterior				
A	4	17.85^a^ (7.14–34.52)	14.46^b∗∗^ (4.52–31.42)	16.54^ab∗∗^ (3.78–32.19)
B	4	14.06^a∗∗∗^ (2.96–34.68)	13.55^ab∗∗∗^ (2.10–23.96)	10.71^b∗∗∗^ (2.20–33.36)
C	2	19.40^†^ (4.83–33.15)	12.58^†^ (5.29–23.38)	13.89^†^ (5.39–26.37)

A: partial thickness tendon tear; B: full-thickness tear with no retraction of tendon; C: full-thickness tendon tear with retraction; PA med.: medial pennation angle; median with ( ) minimum and maximum values. If superscript letters are different, it indicates statistically significant (*P* < 0.001) difference between the superficial, middle, and deep parts of specimens within the same tear category. ^*^ *P* = 0.163 middle and deep; ^**^ *P* = 0.026 superficial and deep, *P* = 0.021 middle and deep; ^***^ *P* = 0.047 superficial and middle, *P* = 0.002 superficial and deep, and *P* = 0.419 middle and deep; *P* = 0.208 middle and deep. ^†^Statistical analysis was not conducted due to sample size.
